# Supplementary material for: Winter is coming–Temperature affects immune defenses and susceptibility to Batrachochytrium salamandrivorans
Source: PLoS Pathog. 2021 Feb 18;17(2):e1009234. doi: 10.1371/journal.ppat.1009234 (PMC7891748; doi:10.1371/journal.ppat.1009234)
Supplement: S1 Table — (DOCX) [file ppat.1009234.s001.docx]

**S1 Table. Percentage mortality, median days survival and results of Kaplan-Meier survival analysis performed on each experiment (Days Survival ~ Dose), between temperature at the same dose (Days Survival ~ Temperature), and between life-stages at the same dose and temperature (Days Survival~Life-stage) for *Notophthalmus viridescens* exposed to *Batrachochytrium salamandrivorans*.**

| **Life Stage** | **Temperature** | **Dose** | **N** | **% Mortality** | **Median Days Survival** | **Days Survival ~ Dose** | **Days Survival ~ Temperature** | **Days Survival ~ Life-stage** |
| --- | --- | --- | --- | --- | --- | --- | --- | --- |
|  |  |  |  |  |  |  |  |  |
| **Adult** | 6 ºC | Control | 5 | 0 | NA | *X^2^*=80.7, *P*<0.001 | 5x10^3^: *X^2^* = 7.1, *P*=0.008 5x10^4^: *X*^2^ = 18.7, *P<*0.001 5x10^5^: *X*^2^ = 15.7, *P*<0.001 5x10^6^: *X*^2^ = 21.5, *P*<0.001 | 6 ºC, 5x10^3^: *X^2^=12.3 , P <*0.001 6 ºC*,* 5x10^4^*: X^2^=*2.8 *, P* =0.09 6 ºC, 5x10^5^: X^2^= 0.3, *P=*0.6 6 ºC, 5x10^6^: X^2^= 14.1, *P<*0.001 14 ºC, 5x10^3^: X^2^=5.6 , *P*=0.02 14 ºC, 5x10^4^: X^2^=8.5 , *P*=0.003 14 ºC, 5x10^5^: X^2^=0.3 , *P*=*0*.6 14 ºC, 5x10^6^: X^2^=4.6 , *P*=0.03 22 ºC, 5x10^6^: X^2^=6 , *P*=0.01 |
|  |  | 5x10^3^ | 10 | 40 | 59 |  |  |  |
|  |  | 5x10^4^ | 10 | 100 | 52.5 |  |  |  |
|  |  | 5x10^5^ | 10 | 100 | 35.5 |  |  |  |
|  |  | 5x10^6^ | 10 | 100 | 17 |  |  |  |
|  | 14 ºC | Control | 5 | 0 | NA | *X^2^*=87.2, *P*<0.001 |  |  |
|  |  | 5x10^3^ | 10 | 90 | 27.5 |  |  |  |
|  |  | 5x10^4^ | 10 | 100 | 23 |  |  |  |
|  |  | 5x10^5^ | 10 | 100 | 16 |  |  |  |
|  |  | 5x10^6^ | 10 | 100 | 8 |  |  |  |
|  | 22 ºC | Control | 5 | 0 | NA | NS |  |  |
|  |  | 5x10^3^ | 10 | 0 | NA |  |  |  |
|  |  | 5x10^4^ | 10 | 0 | NA |  |  |  |
|  |  | 5x10^5^ | 10 | 0 | NA |  |  |  |
|  |  | 5x10^6^ | 10 | 0 | NA |  |  |  |
| **Eft** | 6 ºC | Control | 6 | 0 | NA | *X^2^*=38.6, *P*<0.001 | 5x10^3^: *X*^2^ = 0, *P*=1 5x10^4^: *X^2^* = 8.2, *P*=0.02 5x10^5^: *X^2^* = 25.9, *P*<0.001 5x10^6^: *X^2^* = 26.4, *P*<0.001 |  |
|  |  | 5x10^3^ | 6 | 83.33 | 81 |  |  |  |
|  |  | 5x10^4^ | 6 | 100 | 61.5 |  |  |  |
|  |  | 5x10^5^ | 6 | 100 | 40 |  |  |  |
|  |  | 5x10^6^ | 6 | 100 | 25.5 |  |  |  |
|  | 14 ºC | Control | 5 | 0 | NA | *X^2^*=26.4, *P*<0.001 |  |  |
|  |  | 5x10^3^ | 5 | 60 | 53 |  |  |  |
|  |  | 5x10^4^ | 5 | 40 | NA |  |  |  |
|  |  | 5x10^5^ | 5 | 100 | 16 |  |  |  |
|  |  | 5x10^6^ | 5 | 100 | 9 |  |  |  |
|  | 22 ºC | Control | 5 | 0 | NA | *X^2^*=9.1, *P*=0.01 |  |  |
|  |  | 5x10^4^ | 5 | 0 | NA |  |  |  |
|  |  | 5x10^5^ | 5 | 0 | NA |  |  |  |
