# Supplementary material for: Winter is coming–Temperature affects immune defenses and susceptibility to Batrachochytrium salamandrivorans
Source: PLoS Pathog. 2021 Feb 18;17(2):e1009234. doi: 10.1371/journal.ppat.1009234 (PMC7891748; doi:10.1371/journal.ppat.1009234)
Supplement: S2 Table — (DOCX) [file ppat.1009234.s002.docx]

**S2 Table.** ***Batrachochytrium salamandrivorans* (*Bsal*) copies/uL by life-stage, exposure temperature, and exposure dose (per 10 mL) showing percentage of *Notophthalmus viridescens* infected at necropsy, *Bsal* copies/uL means (x̅), medians (x̃) and standard deviations (SD).**

| **Life Stage** | **Temp** | **Dose** | **% Infection** | **x̅** | **x̃** | **SD** |
| --- | --- | --- | --- | --- | --- | --- |
|  |  |  |  |  |  |  |
| **Adult** | 6 ºC | Control | 0 | NC | NC | NC |
|  |  | 5x10^3^ | 40 | 4840 | 211 | 9398 |
|  |  | 5x10^4^ | 100 | 29928 | 19662 | 27387 |
|  |  | 5x10^5^ | 100 | 166137 | 721 | 414098 |
|  |  | 5x10^6^ | 100 | 37453 | 33190 | 34122 |
|  | 14 ºC | Control | 0 | NC | NC | NC |
|  |  | 5x10^3^ | 90 | 390703 | 379492 | 297156 |
|  |  | 5x10^4^ | 100 | 115606 | 92618 | 94559 |
|  |  | 5x10^5^ | 100 | 168608 | 128620 | 138251 |
|  |  | 5x10^6^ | 100 | 252254 | 279499 | 165789 |
|  | 22 ºC | Control | 0 | NC | NC | NC |
|  |  | 5x10^3^ | 0 | NC | NC | NC |
|  |  | 5x10^4^ | 0 | NC | NC | NC |
|  |  | 5x10^5^ | 0 | NC | NC | NC |
|  |  | 5x10^6^ | 0 | NC | NC | NC |
| **Eft** | 6 ºC | Control | 0 | NC | NC | NC |
|  |  | 5x10^3^ | 66.66 | 25591 | 7093 | 42229 |
|  |  | 5x10^4^ | 100 | 22960 | 20478 | 18119 |
|  |  | 5x10^5^ | 100 | 111600 | 41626 | 143015 |
|  |  | 5x10^6^ | 100 | 557867 | 320852 | 727765 |
|  | 14 ºC | Control | 0 | NC | NC | NC |
|  |  | 5x10^3^ | 100 | 16114 | 7370 | 16430 |
|  |  | 5x10^4^ | 100 | 10782 | 6259 | 13610 |
|  |  | 5x10^5^ | 100 | 155674 | 113451 | 185880 |
|  |  | 5x10^6^ | 100 | 590267 | 419778 | 696694 |
|  | 22 ºC | Control | 0 | NC | NC | NC |
|  |  | 5x10^4^ | 60 | 12 | 6 | 17 |
|  |  | 5x10^5^ | 100 | 25 | 35 | 17 |
|  |  | 5x10^6^ | 83.33 | 36 | 11 | 48 |

NC = Not calculable due to no infections at the specified exposure temperature and dosage.
