# Supplementary material for: Winter is coming–Temperature affects immune defenses and susceptibility to Batrachochytrium salamandrivorans
Source: PLoS Pathog. 2021 Feb 18;17(2):e1009234. doi: 10.1371/journal.ppat.1009234 (PMC7891748; doi:10.1371/journal.ppat.1009234)
Supplement: S3 Table — (DOCX) [file ppat.1009234.s003.docx]

**S3 Table. Tukey multiple comparison of means performed on total proteins recovered from adult *Notophthalmus viridescens* skin between temperatures corrected by body mass (from Fig 6A).**

**
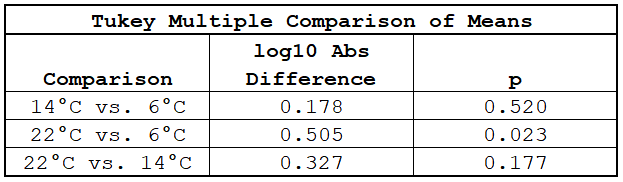
**
