# Supplementary material for: Winter is coming–Temperature affects immune defenses and susceptibility to Batrachochytrium salamandrivorans
Source: PLoS Pathog. 2021 Feb 18;17(2):e1009234. doi: 10.1371/journal.ppat.1009234 (PMC7891748; doi:10.1371/journal.ppat.1009234)
Supplement: S1 Fig — (A) Hydrophobic peptides recovered among temperature treatments corrected for body mass (ANOVA; F2,23 = 0.48, P = 0.62). (B) Percent inhibition of Bsal zoospore viability by concentrated hydrophobic peptides (250 μg/mL) among temperatures when challenged in a growth inhibition assay (F2,14 = 0.99, P = 0.40). (C) Peptide effectiveness (total recovered peptides x percent inhibition of Bsal) among three temperatures (ANOVA; F2,14 = 0.55, P = 0.59). Numbers in brackets indicate the number of N. viridescens sampled. (DOCX) [file ppat.1009234.s004.docx]

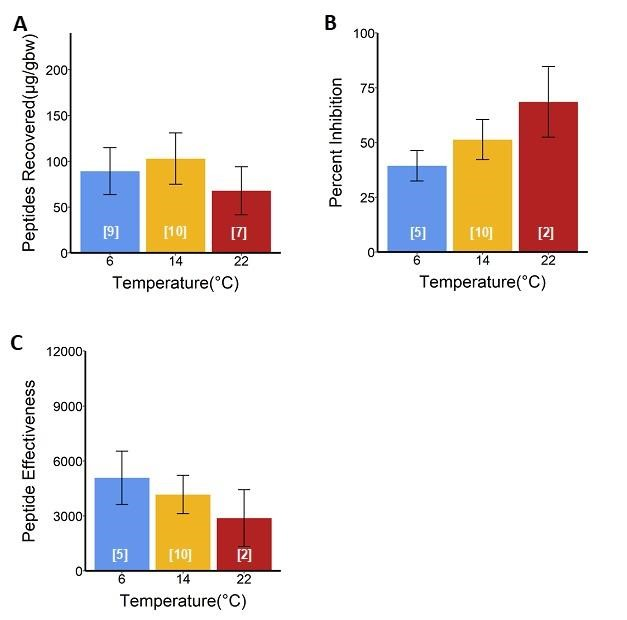


**S1 Fig**. **Putative hydrophobic peptides recovered and inhibition of *Batrachochytrium salamandrivorans* (*Bsal*) zoospore viability by skin secretions from adult *Notophthalmus viridescens* not exposed to *Bsal* (i.e., controls).** (**A**) Hydrophobic peptides recovered among temperature treatments corrected for body mass (ANOVA; F_2,23_ = 0.48, *P* = 0.62). (**B**) Percent inhibition of *Bsal* zoospore viability by concentrated hydrophobic peptides (250 µg/mL) among temperatures when challenged in a growth inhibition assay (F_2,14_ = 0.99, *P* = 0.40). (**C**) Peptide effectiveness (total recovered peptides x percent inhibition of *Bsal*) among three temperatures (ANOVA; F_2,14_ = 0.55, *P* = 0.59). Numbers in brackets indicate the number of *N. viridescens* sampled.
