# Supplementary material for: Winter is coming–Temperature affects immune defenses and susceptibility to Batrachochytrium salamandrivorans
Source: PLoS Pathog. 2021 Feb 18;17(2):e1009234. doi: 10.1371/journal.ppat.1009234 (PMC7891748; doi:10.1371/journal.ppat.1009234)
Supplement: S1 Text — (DOCX) [file ppat.1009234.s005.docx]

**S1 Text. Additional methods on microbiome and *Batrachochytrium salamandrivorans* suitability analyses.**

**Skin Microbiomes: Wild-caught Newts**

In the spring, summer, and fall of 2017, we surveyed adult *Notophthalmus viridescens* in Vermont, USA, to collect general morphometrics and two skin swabs (one for *Bd* detection and quantification and the other for microbiome analysis). Swabbing protocols followed the same methods detailed for the experiments. Microbiome analyses followed the same protocols as described for bacterial communities, with a few minor differences outlined below. First, gDNA was extracted using an IBI Scientific gMAX Mini Genomic DNA Kit (IBI Scientific #IB47282, Dubuque, Iowa USA). We performed PCR reactions in duplicate with the following reaction mixture (25 ul): 10 uL of 2.5X 5 Prime HotStart Master Mix (Quantabio, Beveraly, MA USA), 0.5 uL of each primer (10 uM), 12.0 uL of PCR-grade H20, and 2 uL of extracted DNA template. The PCR cycle conditions followed the Earth Microbiome Protocol 2018. PCR products were purified and equalized using the SequalPrep Normalization Plate Kit (Thermo Fisher Scientific, Cleveland, OH, USA). We followed the same QIIME2 processing detailed previously, except we rarefied at 2000 reads per sample to adequately capture sample diversity and retain the majority of sequenced samples. A PERMANOVA was performed to test for differences in the weighted UniFrac beta diversity microbial community among infection status, between seasons, and with predicted anti-*Bd* function.

***Bsal* Habitat Suitability Modeling**

Our spatial analyses followed Richgels *et al*. (29) except for the modifications below. While Richgels *et al*. (29) used results from *in vitro* growth experiments to inform their ranking scale, we used the results of our *Bsal* temperature susceptibility experiments. Richgels *et al*. (29) scaled temperatures close to 15 ºC as the optimum temperature for *Bsal* resulting in counties with mean annual temperatures and warmest month temperatures closest to 15 ºC receiving a suitability score of four. In the Richgels et al. (29) study, temperatures below or exceeding 15º C were scaled between four and one with temperatures further away from the optimum receiving lower scores.

Based on the results of our adult *N. viridescens* susceptibility trials, we revised the temperature scaling to reflect host-*Bsal* interactions under varying temperatures. At our two highest exposure doses, we found 100% mortality and infection at 6 and 14 ºC exposure temperatures. Therefore, for the habitat suitability model shown in Fig 8, we assumed that exposures between those temperatures will illicit similar infection and mortality responses and therefore provide suitable habitat for *Bsal*. With this assumption, we scaled all mean annual air temperatures and maximum temperatures as four (high infection and mortality) if the value for that raster cell fell between 6 and 14 ºC. Temperatures exceeding 14 ºC were scaled from four to one as they approached 22 ºC. Temperatures exceeding 22 ºC were given a score of one. Temperatures less than our lowest experimental temperature received scores ranging from one to four with temperatures closer to 6 ºC being assigned higher scores. The scores assigned to the annual temperature and maximum temperature values for each raster cell were then averaged to create the *Bsal* habitat suitability score shown in Fig 8.
