# Supplementary material for: Winter is coming–Temperature affects immune defenses and susceptibility to Batrachochytrium salamandrivorans
Source: PLoS Pathog. 2021 Feb 18;17(2):e1009234. doi: 10.1371/journal.ppat.1009234 (PMC7891748; doi:10.1371/journal.ppat.1009234)
Supplement: Attachment — Submitted filename: Responses to Reviewer Comments 11-26-20.docx [file ppat.1009234.s006.docx]

**Responses to Reviewer Comments:**

**NOTE: Line numbers referenced below refer to the “Clean” copy of the revision.**

***********************

Reviewer Comments (if any, and for reference):

Reviewer's Responses to Questions

**Part I - Summary**
Please use this section to discuss strengths/weaknesses of study, novelty/significance, general execution and scholarship.

Reviewer #1: This manuscript reports the results of a rigorous experimental study on an emerging fungal pathogen that constitutes a dramatic threat to salamanders in the Nearctic and Palearctic, threatening to wipe out entire species communities within the next few decades. The results of this study therefore are of high importance as they contribute to our understanding how different salamander and newt species are affected by the pathogen under different environmental conditions, and how the immune system / pathogen interactions are affected by these conditions, in particular temperature.

The study has been thoroughly planned and executed, with a few very minor shortcomings as they are almost unavoidable in such a study with wild-caught animals – e.g., the sampling of different life history stages of the target newt species from different geographic regions. This however does not affect the validity of the results and is openly and adequately discussed in the manuscript.

The author team consists of renowned experts in their field, with a lot of expertise in the study of fungal diseases, microbiomes and protein secretions of amphibians, and I therefore fully trust them to have carried out correctly all the experimental and statistical procedures.

The manuscript is well written and illustrated. I found it a bit annoying that the manuscript does not contain line numbers, this does not help making the work of reviewers easy. I would ask the authors to keep this in mind for their next manuscripts.

**Response**: Line numbers have been added. We apologize – somehow the line numbers were removed during submission.

Reviewer #2: Strengths of this paper are that they conducted a carefully designed experiment and collected relevant response variable data at multiple scales, including cellular histopathology, immune system proteins, microbial communities, pathogen loads, and morbidity/mortality. It is a large, complex and valuable dataset. The topic of this emerging fungal pathogen threat to salamanders in North America is important and urgent.

Weaknesses of this study are that there is not a clear question or hypothesis being put forth, rather the current manuscript aims to see how temperature affects all these things simultaneously. The complex data pieces are not woven together into a coherent narrative. The 'White Walker' bit is funny and catchy, however it is not well explained. What does this really mean for infection dynamics? What do the temperatures that were selected for the experiment actually signify in terms of geography and seasonality? There is an allusion to seasonality and winter, but this is not really explored. Why are both adults and efts being studied separately? These things should all be very clear in the introduction and then fully discussed.

**Response**: We edited the last paragraph of the introduction (lines 101-110) to include hypotheses tested. We expanded our discussion on the geographic and seasonal implications of our results, and moved this information to the beginning of the discussion (lines 239-262). We also elaborated on what it meant by “Winter is Coming” and the “Whitewalker Effect” (lines 255-260, 313). The subsequent paragraphs now only focus on mechanisms, which we feel results in better presentation and greater cohesion.

Reviewer #3: This study presents a large body of careful work on temperature dependent relationships between Bsal pathogenicity, microbiome and antimicrobial protein production. The study hinges on careful newt - infection experiments and these appear to be well done, with adequate sample sizes and attention to dose dependence (often neglected in fungal-infection experiments). The survival analyses appear remarkably coherent in their effect.

Overall, it is hard to fault the experimental basis of the work. The authors could do a better job of contextualise the previous work on temperature and chytrid – the main hypothesis was articulated by Rohr et al and is known as the ‘chytrid-thermal-optimum’ hypothesis: that could be woven in better to set the scene.

Dysbiosis was argued by Bates et al in a recent frontier paper – I think it would be interesting to see whether the taxa noted there are mirrored in this study.

‘The White Walker Effect’ is genius- I’m looking forward to using that metaphor!

**Response**: We understand the reviewer’s point in referencing the “thermal-optimum” and “thermal-mismatch” hypotheses, but we are skeptical of suggesting our research supports either, because we did not specifically design our experiments to test them. That said, we added a few sentences to acknowledge the importance of this previous work with Bd, and recommended future research to test these hypotheses (lines 333-338).

Indeed, understanding pathogen induced changes in the microbiome and whether they lead to dysbiosis as Bates et al as explored, is valuable. Herein, we report on pre-exposure microbiomes, and DCW and MCB will be exploring post-exposure microbiome patterns in an additional manuscript. Such analyses and comparisons to the findings of Bates et al will be explored there, and are beyond the scope of this manuscript.

**Part II – Major Issues: Key Experiments Required for Acceptance**
Please use this section to detail the key new experiments or modifications of existing experiments that should be absolutely required to validate study conclusions.

Generally, there should be no more than 3 such required experiments or major modifications for a "Major Revision" recommendation. If more than 3 experiments are necessary to validate the study conclusions, then you are encouraged to recommend "Reject".

Reviewer #1: (No Response)

Reviewer #2:

1) Re-write the paper to focus on specific questions and hypotheses

**Response:** We revised the Introduction to include hypotheses (lines 101-110) and restructured the discussion to: (1) First, discuss implications of the temperature-dependent susceptibility results (lines 239-262), and (2) discuss the possible mechanisms in the subsequent paragraphs. We also added a paragraph on pathogen replication solely, which previously was mentioned briefly in different paragraphs (lines 263-272). We feel this restructuring increases comprehension of our findings.

2) Conduct an analysis that detangles whether the temperature effects on microbes or immune function is larger/ more important. This may also inform one way to re-structure the intro and frame questions.

**Response**: As per the recommendation of the managing editor, we did not perform additional analyses.

3) who are the microbial players and what are they doing?

**Response**: We added analyses including taxaplots and results describing the main microbioal players, both for bacteria and fungi (see Figure 4 and lines 159-164). We removed the phylogenetic diversity plot for bacteria, but mention that this result is similar to the other alpha diversity metric (sOTU richness) presented. This revision allowed for a balanced figure comparing bacteria and fungi.

Reviewer #3: None identified

**Part III – Minor Issues: Editorial and Data Presentation Modifications**
Please use this section for editorial suggestions as well as relatively minor modifications of existing data that would enhance clarity.

Reviewer #1: Overall, I have only very few suggestions for revision and improvement.

In the Discussion, I think it would be worth mentioning that the experimental temperature regime (constant 6, 14 and 25 °C) only reflects partially the true conditions in the wild, and it would be interesting to carry out experiments with natural temperature regimes, i.e., changes between diurnal and nocturnal temperatures as these newts certainly experience them in the wild. Interestingly, the 6°C temperature regime may quite well reflect the situation during hibernation where amphibians shelter underground in burrows of usually quite constant temperature.

**Response**: We agree that evaluating variation in temperature is the next important step, and acknowledge this in lines 334-338.

To my knowledge, the known cases of salamander dieoffs in the wild in Europe have so far not been related quantitatively to seasons, and in the present study (given its title "Winter is coming" it would be worth providing such a summary. For some time I was convinced most dieoffs of fire salamanders were indeed taking place in the winter, but by now it seems they have been detected in almost all seasons in the year. One interesting point to discuss is also that winter mortality in the wild is very hard to detect as the animals may die within their shelter, thus driving "silent declines" where salamanders could slowly disappear from a region without and detection of mass mortality events.

**Response**: We edited the discussion and provide a potential explanation as to why seasonal trends have not been observed in Europe (although see Beukema et al. 2020). We also cited Buekema’s paper which suggests *Bsal* growth in Europe is linked to seasonality (lines 250-262).

It is awkward that the geographical models are presented only in the Discussion. Especially in the PLOSformat in which methods are given after Results/Discussion, this makes it hard for the reader to understand what these models are about. Two sentences in the Results, briefly describing what was done and what the Results were, referring to Fig. 8, would be helpful.

**Response**: We agree and now present these results in the Results section (lines 229-233).

Other than that, I have only a number of quite minor revisions to suggest, mostly typos and sentences that are difficult to understand and should be rephrased.

Please across the entire manuscript, including Figure captions, make sure to consistently italicize (or not italicize) "Bsal". This is currently very inconsistent and there are many instances of non-italicized occurrences of Bsal.

**Response**: Corrections made throughout manuscript.

Abstract: "emerging fungus" sounds weird, maybe better something like "emerging fungal pathogen"?

**Response**: Suggested edit made (line 34).

Abstract: Please explain at first mention that Nothophthalmus is a newt.

**Response**: We changed the use to “eastern newt” to its scientific name (N. viridescens) throughout the manuscript, which is more consistent with PLOS format.

Results: explain "eft" at first mention.

**Response**: Suggested edit made (line 113).

Figure 2 legend, first line: italicize Bsal and make sure this is consistent throughout the manuscript.

**Response**: We made this edit throughout the manuscript.

Page 6, 1st line: z.ratio or z-ratio?

**Response**: Suggested edit made (line 169).

Page 6, 4th line, a space seems to be missing after period.

**Response**: Suggested edit made.

Page 6, line 15, maybe write "In nature" or "Under natural conditions" rather than "Naturally" which is confusing.

**Response**: We changed to “in the wild” (line 195).

Figure 4 caption, lower case in "microbiome"

**Response**: Suggested edit made (line 181).

Page 8, Skin proteins and peptides, the first sentences are confusing due to the use of the term "greater". I suppose the first use of this term refers to more total protein recovered (= greater recovery = higher concentration, higher amount, more molecules???) and the second use refers to the size of the molecules (greater = larger molecules found)? Please try to rephrase these sentences to make them less prone to misunderstanding.

**Response**: We edited the lines to include the unit of measure being contrasted (line 207-212)

Page 10, " However, total recovered proteins on amphibian skin at 6ºC were greater than 14 and 22ºC," --- please rephrase, probably something like "greater than at 14 ..." would be appropriate.

**Response**: We revised this paragraph (line 286-292).

In the methods, please specify at first mention of swabs that these were skin swabs, and provide details of how these were taken. This methodology is obvious for all chytrid researchers, but maybe not for other readers.

**Response**: Suggested edit made (line 356-357).

There are many (!!) issues with the references. In brief, scientific names are often not italicized, in several references all words start with upper case which is incorrect (e.g., refs. 22, 24, 57...). Journal names are sometimes abbreviated and sometimes not, and sometimes with words not in upper case (e.g., Biological conservation in ref. 30 should be Biological Conservation, ref. 27 should be Trends in Ecology & Evolution, and so on). These issues are too numerous to be listed here, the authors really should check them one by one and make sure to have them corrected in the reference management system.
Is it really PLOS format to abbreviate page ranges (e.g., 1627-39 rather than 1627-1639)? – I have not seen this in multiple PLOS papers I checked.

**Response**: We checked all references, made edits, and ensure they followed PLOS Pathogens format. (References section).

Reviewer #2: Overall, the writing quality is fine.

Reviewer #3: The authors are commended for including fungal ITS into the 'microbiome' however don't discuss what appears to be quite divergent patterns. Perhaps that could be explored a little more.

**Response**: We have expanded the discussion to include a description of the taxa (lines 159-164, Figure 4), and exploration of alpha diversity results indicating greater diversity of bacteria at 14C and greater diversity of fungi at 22C (lines 165-169). We suggest that competition between these groups is possible (lines 274-281).

PLOS authors have the option to publish the peer review history of their article ([what does this mean?](https://journals.plos.org/plospathogens/s/editorial-and-peer-review-process#loc-peer-review-history)). If published, this will include your full peer review and any attached files.


If you choose “no”, your identity will remain anonymous but your review may still be made public.


**Do you want your identity to be public for this peer review?** For information about this choice, including consent withdrawal, please see our [Privacy Policy](https://www.plos.org/privacy-policy).

Reviewer #1: No

Reviewer #2: No

Reviewer #3: No
